# Synthesis, Characterization, and Antimicrobial Properties of Peptides Mimicking Copolymers of Maleic Anhydride and 4-Methyl-1-pentene

**DOI:** 10.3390/ijms19092617

**Published:** 2018-09-04

**Authors:** Marian Szkudlarek, Elisabeth Heine, Helmut Keul, Uwe Beginn, Martin Möller

**Affiliations:** 1 DWI Leibniz Institute for Interactive Materials and Institute of Technical and Macromolecular Chemistry, RWTH Aachen University, Forckenbeckstraße 50, D-52056 Aachen, Germany; szkudlarekmh@gmail.com (M.S.); keul@dwi.rwth-aachen.de (H.K.); 2 Institut für Chemie, Universität Osnabrück, OMC, Barbarastraße 7, D-49076 Osnabrück, Germany

**Keywords:** cationic polymers, imidization, quaternization, antimicrobial properties, hemolytic activity

## Abstract

Synthetic amphiphilic copolymers with strong antimicrobial properties mimicking natural antimicrobial peptides were obtained via synthesis of an alternating copolymer of maleic anhydride and 4-methyl-1-pentene. The obtained copolymer was modified by grafting with 3-(dimethylamino)-1-propylamine (DMAPA) and imidized in a one-pot synthesis. The obtained copolymer was modified further to yield polycationic copolymers by means of quaternization with methyl iodide and dodecyl iodide, as well as by being sequentially quaternized with both of them. The antimicrobial properties of obtained copolymers were tested against *Escherichia coli*, *Pseudomonas aeruginosa*, *Staphylococcus epidermidis*, and *Staphylococcus aureus.* Both tested quaternized copolymers were more active against the Gram-negative *E. coli* than against the Gram-positive *S. aureus.* The copolymer modified with both iodides was best when tested against *E. coli* and, comparing all three copolymers, also exhibited the best effect against *S. aureus*. Moreover, it shows (limited) selectivity to differentiate between mammalian cells and bacterial cell walls. Comparing the minimum inhibitory concentration (MIC) of Nisin against the Gram-positive bacteria on the molar basis instead on the weight basis, the difference between the effect of Nisin and the copolymer is significantly lower.

## 1. Introduction

Since in 1960s, Cornell and Donaruma [1] have described 2-methacryloxytropones-based polymers that exhibited antibacterial activity, and antimicrobial copolymers have since gained significant interest. Over the last decades, a large number of publications and multiple reviews exploring antimicrobial polymers have been published. In the 1990s and at the beginning of 21st century, the main focus was on the synthesis and chemical nature of the polymers [2]. For example, Kenawy et al. [3] classified antimicrobial copolymers regarding their structure as quaternary ammonium salts (QAS) [4,5,6,7,8,9,10,11,12,13,14,15,16,17,18,19,20], phosphonium salts [20,21,22,23,24,25,26,27], sulfonic acid derivatives (salts, sulfonamides), and N-halamines. In most recent reviews, the main focus has been on the bactericidal mechanism and influence of relevant parameters such as molecular weight and charge distribution [28]. The antimicrobial properties of maleic anhydride copolymers with olefins [29] and styrene [30,31,32] have been investigated since the 1960s. In all cases, succinic anhydride functionality was used as a base for further modification in order to introduce an antibacterial moiety (diamine, aminophenol, etc.). From this perspective, the modified maleic anhydride copolymers belong to one of the groups mentioned above. These polymers are usually amphiphilic and act as surfactants.

At the same time, antimicrobial peptides represent a large group of natural compounds with a broad spectrum of antimicrobial activity [33,34,35,36]. The antimicrobial activity of these molecules also comes from their amphiphilic structure [34].

In the past, much effort has been made to understand the mechanism of cytotoxicity of amphiphilic peptides. One of the examples is Melittin from bee venom, which has the highest hemolytic activity. Compared to Magainin from *Xenopus* frog skin, it has a high content of hydrophobic residues. It has a 26-amino-acid-residue-long sequence with a characteristic cluster of lysine and arginine residues. Magainins, 23-residue-long peptides, are essentially toxic for bacterial strains while being poorly hemolytic [37]. Synthetic peptides of different composition and structure have been widely investigated for their antimicrobial properties. Particular attention has been paid to peptides, which are only constituted by nonpolar leucine and charged lysine, so-called LK-peptides [37,38]. These molecules are ideally amphiphilic and proper choice of the Lys:Leu ratio as well as the chain length enables designing structures most similar to natural toxins.

With the aim to design an optimum antimicrobial polymer and to set up a structure–function relationship, many groups, including ours, have constructed amphiphilic polymers with different backbones and specific cationic and hydrophobic residues in defined ratios mimicking antimicrobial peptides. An overview on the peptide mimetic design of antimicrobial polymers can be found, e.g., in [39]. Specific design can lead to broad-spectrum antibacterials or polymers with Gram-selectivity [40].

**Scheme 1:**
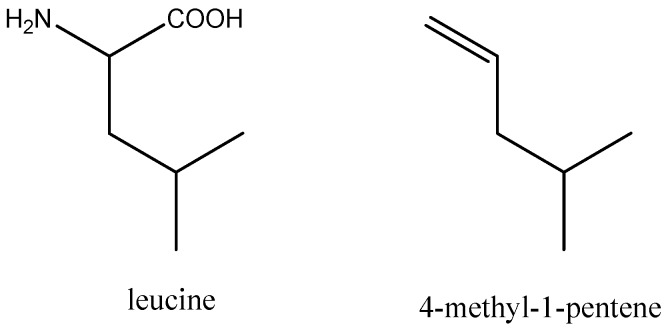
Comparison of the structure of leucine with 4-methyl-1-pentene.

The aim of creating a new synthetic, amphiphilic structure with strong antimicrobial properties comparable to natural antimicrobial peptides led to the choice of an alkene and maleic anhydride copolymer. Lower alkenes and maleic anhydride yield alternating copolymers. This ensures a constant 1:1 ratio of the hydrophobic and cationic part similar to those in Leu:Lys 1:1 LK-peptides. The choice of 4-methyl-1-pentene as a hydrophobic comonomer is based on the similarity of its structure with leucine (cf. Scheme 1), while the choice of maleic anhydride leaves ample space for further design of the hydrophilic part by means of chemical modification.

This work covers polymeric quaternary ammonium salts obtained by chemical modification of maleic anhydride 4-methyl-1-pentene copolymers. The free radical copolymerization of maleic anhydride with 4-methyl-1-pentene leads to alternating copolymers, which were grafted using *N*,*N*-substituted diamines. Although the antimicrobial activity of nonquaternized *N*,*N*-substituted derivatives has been reported [1], biocidal activity should be enhanced by quaternization of the tertiary amine groups with different alkyl halides. The higher biological activity of positively charged moieties can be explained by electrostatic interaction with negatively charged cell surfaces. The advantage of polycations is based on the higher charge density that allows highly extended adsorption on the bacterial cell. The expected penetration of the cell wall and disruption of the membrane by the lipophilic alkane chain releases cytoplasmic constituents and leads to the death of the cell.

## 2. Results and Discussion

### 2.1. Copolymerization of Maleic Anhydride with 4-Methyl-1-pentene

Alternating copolymers of maleic anhydride with alkenes were obtained by free-radical copolymerization in solution in the presence of radical initiators [41,42,43,44,45]. However, copolymers which contain an excess of olefin have also been described in the literature [46,47], but as a general rule, alternating copolymers are formed, in particular, when an excess of anhydride was used.

**Scheme 2:**
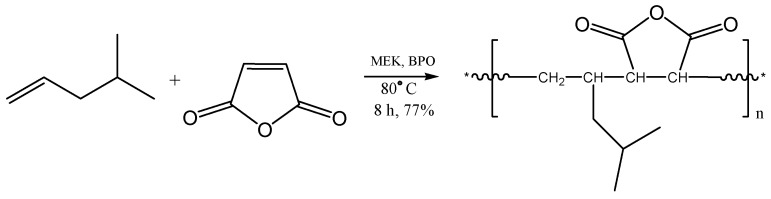
Copolymerization of maleic anhydride (MSA) with 4-methyl-1-pentene.

The alternating copolymer of 4-methyl-1-pentene with maleic anhydride (MSA) was synthesized accordingly via free-radical polymerization. The copolymerization was carried out under homogenous conditions in anhydrous 2-butanone (MEK) at 80 °C in the presence of benzoyl peroxide (BPO) as an initiator. Scheme 2 depicts the copolymerization reaction of 4-methyl-1-pentene with maleic anhydride.

An excess of maleic anhydride was used to ensure the equimolar composition of the resulting copolymer. The product was separated from the reaction mixture by precipitation in a Et_2_O:MeOH (4:1, vol:vol) mixture. For its molecular weight determination by gel permeation chromatography (GPC), the succinic anhydride units of the copolymer were methanolized at room temperature. The methanolysis was necessary because maleic anhydride copolymer adsorbed on the inline filter, impeding any measurement. GPC measurement of the methanolyzed copolymer was performed in THF with PMMA standards and the determined values were: M_n_ = 5800 and M_w_/M_n_ = 1.67.

**Figure 1:**
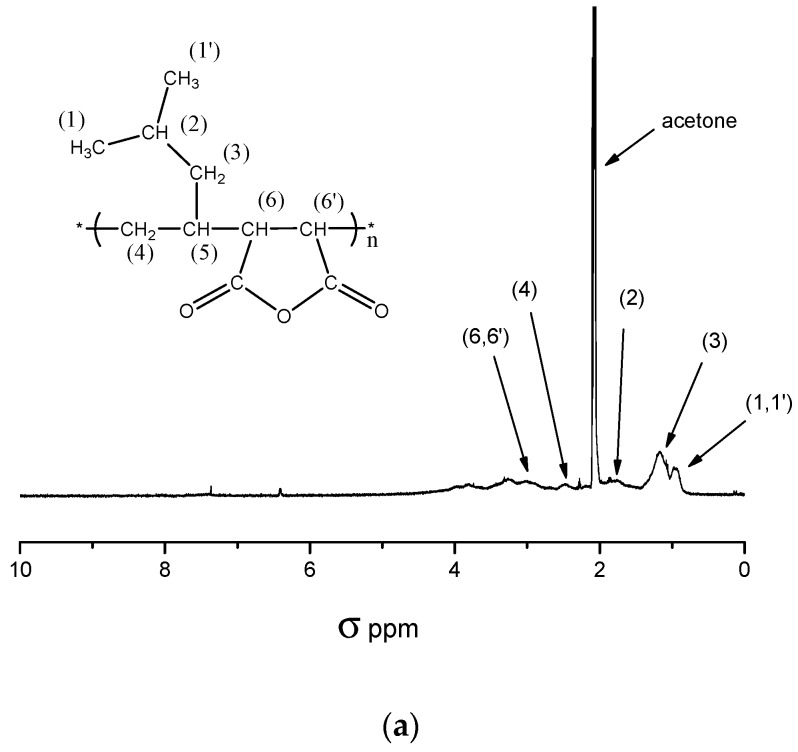

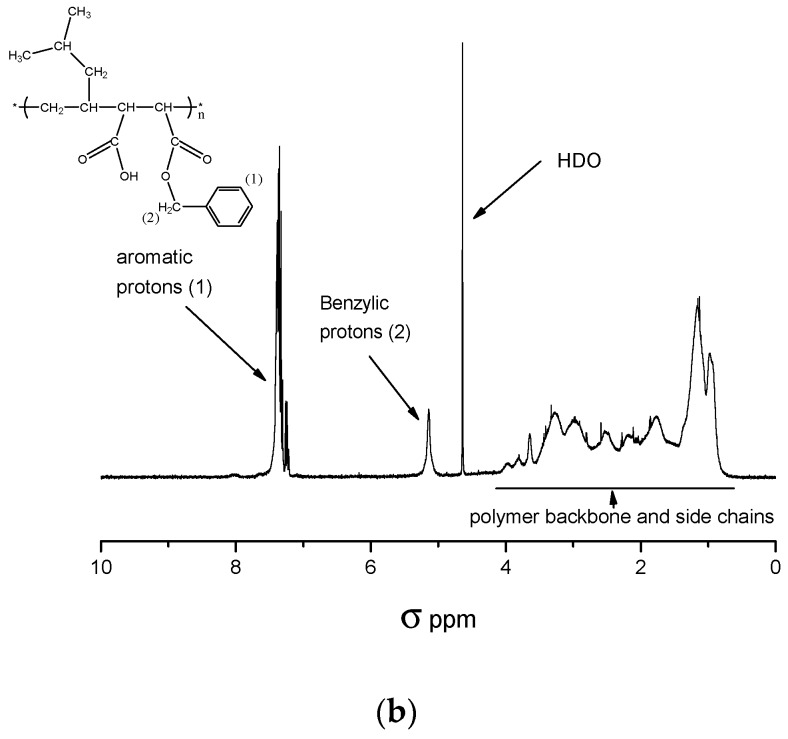
(**a**) ^1^H-NMR spectrum of P[MP-alt-MSA] measured in acetone d_6_; (**b**) ^1^H-NMR spectrum of P[MP-alt-MSA] esterified with benzyl alcohol measured in D_2_O.

The composition of the obtained polymer could not be determined by ^1^H-NMR because of overlapping signals of both monomers. Figure 1a depicts a typical ^1^H-NMR-spectrum of a P[MP-alt-MSA] copolymer. The range of overlapping signals between 1 and 4 ppm precludes the calculation of the polymer composition for the non-methanolyzed copolymers (MSA protons 3.25 ppm) as well as methanolyzed products (MSA protons 2.8 ppm) [47].

Treatment of the copolymer with benzyl alcohol in MEK results in the formation of monobenzyl esters. This method allows determining the content of protons of succinic acid benzyl ester since the benzyl group’s NMR signals were well separated from the other polymer peaks. Figure 1b depicts the ^1^H-NMR spectrum of a P[MP-alt-MSA] copolymer after reaction with benzyl alcohol. The aromatic protons as well as the benzylic protons are well separated from the signals of the polymer backbone and the alkyl side groups. The monomer composition was calculated from the integrated signal intensity of the benzylic protons and that of the alkyl signals between 0.8 and 5.2 ppm according to Equation (1):

(1) $$F_{MSA\;}=\frac{\frac{A}{m}}{\frac{A}{m}+\frac{B}{n}}$$

*A*—integration value of benzylic proton signal (*σ* = 4.90–5.30 ppm),
*m*—number of benzylic protons = 2,
*B*—integration value of polymer backbone and side chains proton signal (*σ* = 0.70–4.2),
*n*—number of protons in signal mentioned in *B* = 14.

This method was developed for determination of the MSA content in terpolymers, and it was proved that, without a catalyst, only one carboxyl group was esterified [48]. It is very useful and precise in the range of error of the used spectroscopic method. The 46 ± 5% of calculated content of succinic anhydride in the copolymer is reasonable and allowed us to assume that an alternating copolymer was obtained. The composition of the copolymer was also determined by elemental analysis. The obtained results correspond well to the composition calculated for alternating copolymer (see Table 1).

### 2.2. Grafting of DMAPA onto P[MP-alt-MSA] Copolymer

#### 2.2.1. Model Reaction of Amidoacidification

**Scheme 3:**
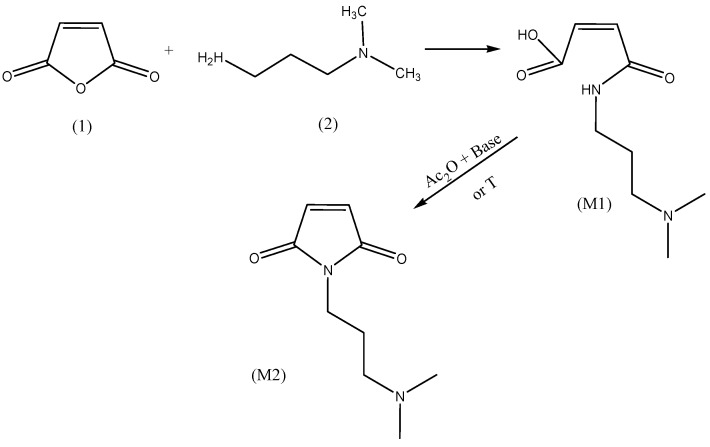
Model reaction of amidoacidification and imidization of maleic anhydride with -(dimethylamino)-1-propylamine (DMAPA).

Maleic anhydride can easily react with 3-(dimethylamino)-1-propylamine (DMAPA) to yield 3-(*N*,*N*-dimethylamino)propyl maleamic acid (M1). The reaction was carried out in chloroform by addition of the amine to MSA at room temperature and subsequent heating to 60 °C. The reaction is exothermic and proceeds easily, hence, the heating step was performed to ensure complete reaction. The product was precipitated in a fivefold excess of acetone and dried. The desired compound has been obtained as a white powder. Scheme 3 depicts the reaction of maleic anhydride with DMAPA.

**Figure 2:**
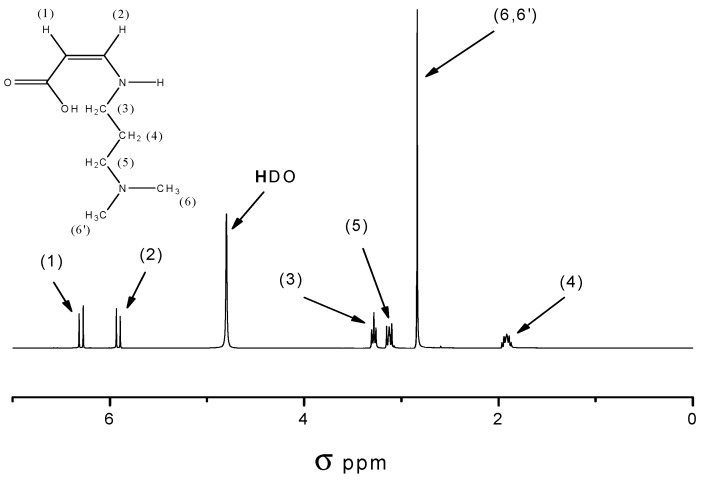
^1^H-NMR spectra of cis-3-(*N*,*N*-dimethylamino)propyl maleamic acid.

Because of its molecular asymmetry, the product can be easily identified and distinguished from nonreacted MSA by means of ^1^H-NMR. In the course of the reaction, the single peak of MSA around 7 ppm (double bond protons) disappeared and two doublets appeared at 6.33 and 5.96 ppm, respectively (see Figure 2).

#### 2.2.2. Amidoacidification and Imidization of Poly[(4-methyl-1-pentene)-alt-maleic Anhydride

**Scheme 4:**
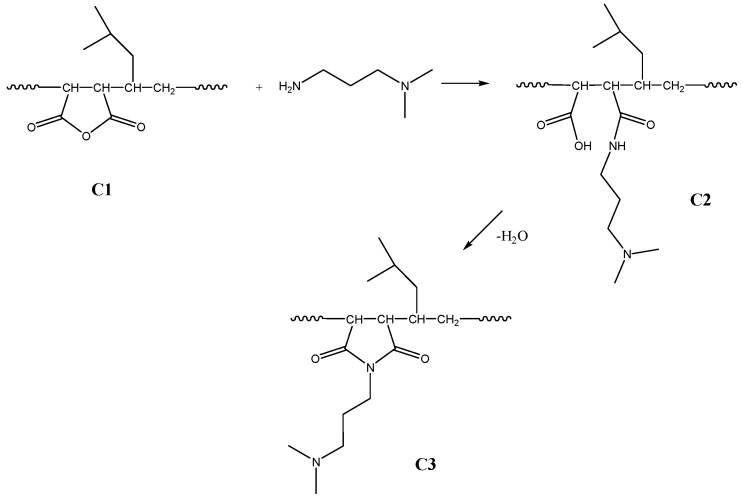
Amidoacidification and imidization of MSA copolymer.

Scheme 4 depicts the reaction of the anhydride moiety in the polymeric chain (C1) with DMAPA, yielding amidacid (C2), and followed by thermal imidization, which yields the cyclic N-substituted imide (C3).

The amidoacidification of maleic anhydride copolymer with *N*,*N*-dimethylamino-1-propyl amine (DMAPA) was performed analogously to the model reaction [18,44,49,50,51,52,53,54,55,56,57,58,59,60]. The amine was added dropwise to a dimethylformamide DMF solution of the copolymer. The product precipitated from the reaction mixture and could be easily obtained in its pure form. Because of the heterogeneity of the reaction mixture, an excess of amine was used and the mixture was stirred for about 20 h to obtain an almost quantitative conversion. This intermediate product was separated by filtration and analysed.

**Figure 3:**
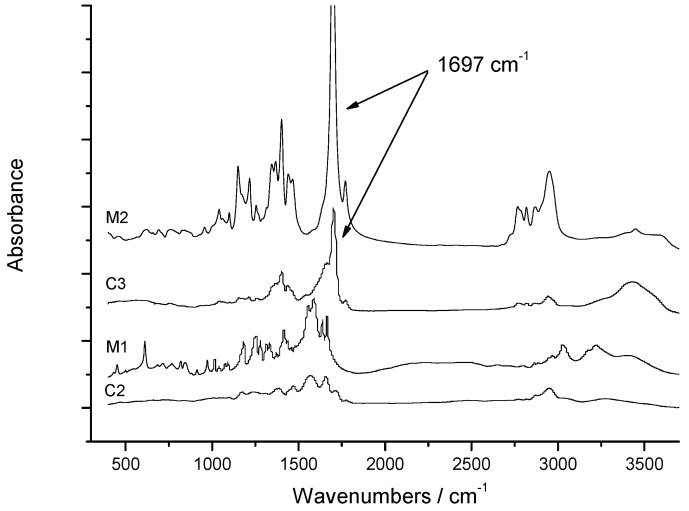
IR spectra of amidoacidified C2, thermally imidized C3, and model compounds M1 and M2 (3-(*N*,*N*-dimethylamino)propyl maleamic acid and 3-(*N*,*N*-dimethylaminopropyl)maleimide). The characteristic absorption band of cyclic imides in C3 and M2 at 1697 cm^−1^ are shown.

The prepared macromolecular amic acid could not be characterized well by means of NMR spectroscopy because of the occurrence of broad overlapping signals. The IR spectra of the amidoacidified copolymer C2 shown in Figure 3 corresponded to the comparative spectrum of the model product (Figure 3 M1) from the reaction of maleic anhydride with DMAPA.

To determine the content of modified succinic anhydride moieties, elemental analysis was employed. The standard measurement for carbon, hydrogen, and nitrogen determination showed a strong correlation to the calculated values for the hydrogen and nitrogen content while measured content of carbon strongly differed from the calculated value (Table 2). However, the product was found to be hygroscopic [50] and the standard analytical treatment did not include a drying step. When one recalculates the elemental composition of the copolymer assuming that each amic acid unit additionally binds two molecules of water, the determined elemental composition becomes reasonable (see Table 2, #3).

The abovementioned hypothesis was verified in a simple experiment. A small sample of the C2 was dried in vacuo to a constant weight and placed in an open vessel on a very precise balance to absorb the humidity from air. A rapid increase in weight of the sample was observed. The calculated difference between initial mass of the sample and the mass after 12 h of exposure to humid air showed that the composition of the copolymer was C_15_H_28_N_2_O_3_·1.86H_2_O. The difference between the expected and obtained values can be explained as a result of insufficient drying, absorption of water during transfer from the drying oven to balance (the initial period of the experiment showed a rapid increase of the weight), degree of modification lower than assumed 100%, or impurities. Most probably it should be treated as the combination of several reasons, although the measured value of ~1.9 moles H_2_O per amic acid unit is very close to the postulated stoichiometric 1:2 composition.

#### 2.2.3. Chemical Imidization

The imidization reaction of amic acid in the presence of a dehydration agent and a base has been described in many publications [51,52,58,59,60]. The most common reagents are acetic anhydride in the presence of triethylamine or sodium acetate. This reaction is usually carried out under mild conditions (temperature 80–90 °C) and is useful for the synthesis of a wide range of N-substituted imides.

The reaction was first tested by means of a model reaction between cis-3-(3-dimethylaminopropyl carbamoyl) acrylic acid and acetic anhydride. As depicted in Scheme 3, the substrate (M1) was heated at 90 °C for 4 h in the presence of acetic anhydride (dehydrating agent) and sodium acetate (catalyst).

At the end of the reaction time, the mixture was cooled down and sodium acetate was separated by filtration. Acetic anhydride, as well as acetic acid formed during the reaction, were distilled off under reduced pressure. The product was obtained as a brown oily liquid. 

**Figure 4:**
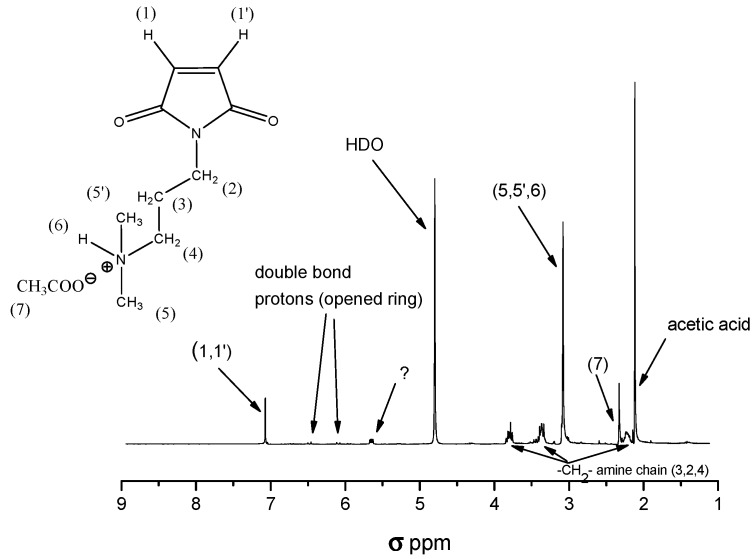
^1^H-NMR spectra of crude 3-(3-dimethylaminopropyl)maleimide. Characteristic peak at ~7 ppm confirms the ring formation.

^1^H-NMR analysis of the crude 3-(3-dimethylaminopropyl)maleimide (see Figure 4) shows trace signals of double-bond protons (between 6 and 6.5 ppm) from unreacted maleic acid amide, which is about 2% in respect to maleimide.

However, the singlet at ~7 ppm shows that the five-membered ring has been successfully formed. The comparison between the signals at *σ* = 7 ppm and aliphatic protons (*σ* = 2–4 ppm), which belong to the amine chain, shows that less than 50% of the material that has been obtained was converted to the required compound. The excess of aliphatic amine protons cannot be explained by incomplete conversion of the amic acid because of the very low signal intensity of its double-bond protons. Also, a very strong signal of acetic acid has been registered, although the sample was kept under vacuum for long time.

The IR spectrum (Figure 3) shows the characteristic absorption bands of cyclic imides at 1697 and 1714 cm^−1^ that confirm the imide formation.

The reaction of amidoacidified C2 in the presence of Ac_2_O and both mentioned bases always yielded dark brown products of sometimes even tarry consistency. Although characteristic absorption bands of imides were observed, no way was found to purify the macromolecular product or at least to remove the dark color.

#### 2.2.4. Thermal Imidization

Because of the poor quality of obtained products, other possibilities of imidization were explored. Thermal imidization of amic acids as a main synthesis method has been described in many organic chemistry handbooks and publications [18,53,54,55,56,57,58].

**Figure 5:**
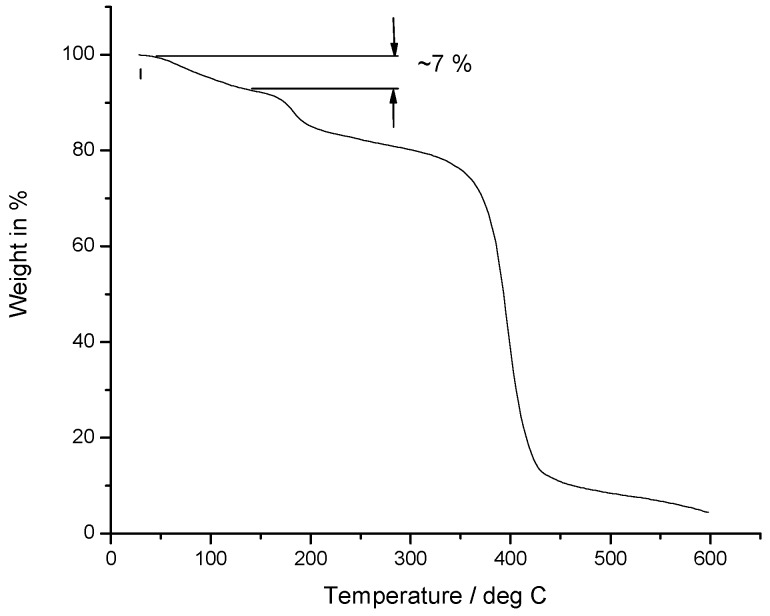
Thermogravimetric analysis (TGA) measurement of amic acid form C2.

TGA analysis of copolymer 2 was performed in order to determine suitable conditions for the reaction. The calculated mass difference between amic acid form of the copolymer and its imidized form was 6.5%. The thermogravimetric analysis showed a weight loss of approximately 7% in the temperature range between 80 and 130 °C, which was attributed to the formation of the cyclic imide by elimination of water (see Figure 5).

The imidization of C2 was carried out in DMF at 120 °C. The imidized copolymer C3 was obtained in form of a pale cream-colored powder which was soluble in acetone. For comparison, the amidoacidified C2 was insoluble in acetone. The infrared spectroscopy (see Figure 3) showed a characteristic absorption band of imides at 1567 cm^−1^.

The advantage of thermal imidization is its simplicity and the improved product quality as well as the absence of other reagents. It is of paramount importance for an antibacterial test to avoid the presence of low-molecular-weight toxic agents, which can cause false positive results.

The molecular weight of the imidized copolymer was measured by THF-GPC. The measurement showed an increase of the molecular weight of the copolymer after modification from M_n_ = 5800 to M_n_ = 8800. This increase in M_n_ is partially caused by the modification as well as by removal of the low molecular weight fraction during purification. This is indicated also by a change of the polydisperisity of the sample from M_w_/M_n_ = 1.67 to M_w_/M_n_ = 1.47.

### 2.3. Quaternization of C3

The quaternization reaction was carried out in solution according to the method described in the literature [18,50,53,54,55,61,62,63]. The C3 copolymer was dissolved in DMSO and reacted with methyl iodide to give copolymer C4. With a mixture of methyl iodide and dodecyl iodide (1:1, mol:mol), copolymer C5 was obtained and applying dodecyl iodide alone yielded copolymer C6. The reaction was performed at room temperature in the presence of an excess of alkyl iodide (with respect to amine groups), yielding quantitative conversion of the tertiary amine.

According to the literature, a long alkyl chain within the ammonium group shows better biocidal properties [2,64,65]. For this reason, three different quaternized polymers have been prepared bearing trimethyl ammonium, dimethyl-dodecyl ammonium groups, as well as a 1:1 (mol:mol) mixture of both ammonium moieties as the quaternary ammonium side groups.

The IR spectra of quaternized copolymers showed no significant changes because the absorption bands characteristic for ammonium salts overlap with absorption bands of the nonquaternized copolymer (~1500 and 3000 cm^−1^).

### 2.4. Solubility in Selected Solvents

The different polarity of the copolymers C3, C4, C5, and C6 is also reflected in the different solubility (see Table 3).

The introduction of cationic moieties into the polymeric chains strongly increases the polarity of the copolymer and has a huge influence on the solubility. While nonquaternized copolymer C3 was well soluble in all of the chosen solvents, the methyl-iodide-quaternized copolymer C4 was soluble only in very polar solvents such as water and DMSO but insoluble even in lower alcohols such as methanol and ethanol. The introduction of long alkyl chains by quaternization with dodecyl iodide makes copolymer C6 insoluble in water, DMSO, DMF, and alcohols but well soluble in ketones, THF, and chloroform. The simultaneous quaternization with both methyl iodide and dodecyl iodide (C5) ensures good solubility in very polar solvents (water, DMSO) as well as less polar as 2-butanone. The solubility of the quaternized copolymers in water is of utmost importance for antimicrobial investigations.

### 2.5. Thermal Properties

**Figure 6:**
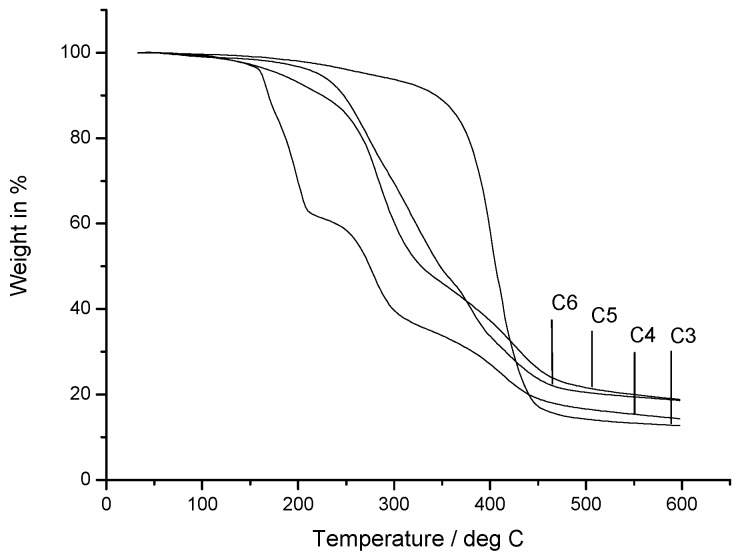
TGA measurement of nonquaternized C3 and quaternized copolymers: C4 (-N(CH_3_)_3_^+^), C5 ((-N(CH_3_)_3_^+^ + -N(CH_3_)_2_ C_12_H_25_^+^), and C6 (-N(CH_3_)_2_ C_12_H_25_^+^).

Figure 6 depicts the TGA thermograms of copolymer C3 and its quaternized derivatives C4, C5, and C6. The nonquaternized copolymer C3 (curve 1) is the thermally most stable polymer and shows only slight weight loss below 200 °C. The highest weight loss is observed above 300 °C. Any modification of C3 by quaternization causes a decrease in thermal stability of the polymer due to Hofmann elimination of the ammonium groups [61]. The Hofmann elimination occurs when quaternary ammonium salts are exposed to high temperatures and the reaction yields an alkene and a tertiary amine and a low-molecular-weight compound specific for the counterion (e.g., water, HCl, HI, etc.).

The quaternization with methyl iodide causes the C4 (curve 2) to decompose above 150 °C via a three-stage thermal degradation. The first stage starts at 150 °C and ends at 200 °C with a weight loss of 32% corresponding to the loss of HI (31 wt %). Even partial quaternization with long alkyl-chain iodide versus methyl iodide increased the thermal stability, as observed in curves 3 and 4 (C5 and C6) in Figure 6

However, such derivatives also show faster thermal degradation than nonquaternized C3. In all cases, the investigated copolymers showed a certain weight loss at relatively low temperatures around 100 °C, most probably caused by a loss of adsorbed water because of the hygroscopic nature of salts. 

DSC measurement of dodecyl-iodide-quaternized copolymer is typical for all ammonium copolymers based on the C3 copolymer in the temperature range of −50 to 200 °C. Each copolymer showed two thermal transitions: one at 17 °C and a second one in the region of 80–105 °C (see Table 4). The temperature of the second transition seems to depend on the type of quaternizing agent and is about 20 °C higher for copolymers quaternized with dodecyl iodide. There is a correlation with the TGA data where the presence of dodecyl iodide increased the thermal stability. Since the copolymers undergo decomposition in this range of temperatures, reverse heating does not reproduce the curves. Only the first transition is fully reproducible. The fact that the presence of longer alkyl chain gives a higher *T_g_* value is not in line with the expectations. It is known that polymers which contain longer alkyl side chains exhibit lower glass transition temperatures than the shorter ones due to the plastifying effect. Although there has been no melting temperature observed, this phenomenon can be assigned to the formation of ordered structures on the micro or even nano scale. These types of crystalline structures usually do not give any measurable thermal response and further investigation is required. Detailed values of the thermal transition of the investigated copolymers are summarized in Table 4.

### 2.6. Investigation of Antimicrobial Properties of the Cationic Copolymer

In the World Health Organization’s (WHO) “global priority list of antibiotic-resistant bacteria to guide research, discovery, and development of new antibiotics” of February 2017, experts agreed on grouping pathogens according to the species and the type of antibiotic resistance and classified the results in three priority tiers: critical, high, and medium. 

Our selected bacteria belong to genera that were grouped into the priorities critical and high, i.e., here, the prevalence of resistance against antibiotic treatments is extremely high. With our research field, to prepare antimicrobially functional polymers, we would like to contribute to the reduction of the spread of these bacteria.

Amphiphilic polymers with quaternary ammonium groups are known to have antimicrobial properties [20]. Thus, the water soluble C3, C4, and C5 were tested for their antimicrobial efficacy.

In order to find the MIC of the copolymers, bacterial growth of strains belonging to clinically relevant genera was monitored in the presence of all the copolymers. The tests were performed in microwell plates and the proliferation potential of the bacteria was monitored at 37 °C by measuring the optical density at 612 nm for 20 h using a microwell plate incubator/reader in comparison to a reference without the respective polymer. The MIC values are summarized in Table 5.

The novelty of this paper is (i) the polymer backbone based on maleic anhydride and 4-methyl-1-pentene and (ii) the fact that the results obtained for *E. coli* and *S. aureus* were not as expected based on a great amount of papers showing that amphiphilic polymers with different backbones and manifold kinds of cationic and hydrophobic residues are more active against Gram-positive bacteria (lower MIC values) than against Gram-negative bacteria.

The polymers are more effective by a factor of 5–10 against the Gram-negative *E. coli* than against the Gram-positive *S. aureus*. This selectivity, although with a lower factor, was also found in [66], who prepared lysine and arginine mimicking amino and guanidine propyl methacrylamide copolymers; in the latter, the amine-containing copolymers were best.

Compared to polymer C4 (modified with methyl iodide), polymers C3 (nonquaternized) and C5 (modified with both iodides, methyl and dodecyl iodide) are more active against the Gram-negative bacteria *E. coli* and *P. aeruginosa*, whereas, in the case of *S. aureus*, C5 was the most active polymer compared to C3 and C4, with the nonquaternized C3 being the least active in the latter case. Previous studies in our group, although with a different polymer backbone, have shown that the best results against *S. aureus* were obtained with the cationic residue directly linked to the aliphatic residue without a spacer in between [67], and that longer alkyl chains showed best efficacy [68] and, thus, confirm these results. Regarding the solubility properties (Table 3), C3 and C5 show similar solubilities in the different solvents compared to C4. The limited solubility of the latter seems to restrict the effect against *E. coli*, *P. aeruginosa*, and *S. aureus* since the hydrophilic–lipophilic balance is decisive for the efficacy of the respective polymer.

Against *S. epidermidis*, all three polymers are equally active in a comparable range, such as C3 and C5 against *E. coli*.

Comparing the MIC for the Gram-negative bacteria gave 5- (C3) to 20-fold (C5) higher values for *P. aeruginosa* than for *E. coli*, meaning that *E. coli* is 5–20 times better inhibited compared to *P. aeruginosa*. Gram-negative bacteria are known to actively secrete outer membrane vesicles (OMVs) from the outer membrane (OM) [69]. OMV production is correlated with an increased rate of survival upon antimicrobial peptide treatment [70] In *Pseudomonas putida*, OMVs are generated, e.g., as a response to stress caused by cationic surfactants which can contribute to OMV biogenesis, through a physical mechanism by induction of the curvature of the membrane [71]. Although OMV production is common in many bacteria, the extent and mechanism of OMV production is species specific, and thus, the higher MIC values for *P. aeruginosa* might be due to the level of OMV production, since environmental stresses result in increased OMV formation by *P. aeruginosa* [72].

For the Gram-negative bacteria *E. coli* and *P. aeruginosa* and for the Gram-positive *S. aureus*, C5 quaternized with the long alkyl chain, i.e., the repeat unit structure with the hydrophobic moiety being directly accompanied by the charged moiety, exhibits a higher efficacy compared to C4 quaternized with methyl iodide (this was also confirmed for functionalized polymers with polglycidol backbone [73]).

C5 exhibits a more ordered structure due to phase separation and orientation of the hydrophobic C_12_H_25_ chains to hydrophobic domains (see also the discussion on the thermal properties in Section 2.5).

Whereas polymers C3 and C4 led to an agglutination of human RBC at all concentrations tested (10–1000 µg/mL), C5 did not agglutinate RBC but showed lysis of 50% of the RBC relative to the positive control (HC_50_) at a concentration of 60 µg/mL. The higher value of HC_50_ compared to the MIC_100_ against *E. coli* (10 μg/mL) proved that polymer C5 has a selectivity to differentiate between mammalian cells and bacterial cell walls. However, since the values are overall in the same order of magnitude, the selectivity is low.

Since the investigated antimicrobial copolymers were designed to mimic peptides, a comparison with a reference compound is needed. The type A lantibiotics, e.g., Pep5 or Nisin, are in general of linear conformation and all the Nisin type peptides are positively charged [33]. Combination of the cationic nature and the presence of leucine makes Nisin a good reference.

Nisin is a 34-residue-long peptide which is predominantly active against Gram-positive bacteria (Nisin is also active against Gram-negative bacteria but only after a pretreatment) [74]. It is generally accepted that the bacterial plasma membrane is the target for Nisin, and that Nisin kills the cells by pore formation and inhibition of peptidoglycan synthesis. The pore formation causes collapse of vital ion gradients, resulting in cell death [75]. In this study, it was shown that Nisin, compared to the copolymer modified with both iodides, is highly active against Gram-positive bacteria, and, as expected, 30 to more than 60 times less active against Gram-negative bacteria. Moreover, comparing the MIC of Nisin against the Gram-positive bacteria on the molar basis instead on the weight basis, the difference between the effect of Nisin and the copolymer is significantly lower.

This gap indicates that the activity of the peptides is determined not only by the amphiphilic nature but most probably the secondary peptide structure plays also a substantial role.

## 3. Materials and Methods

### 3.1. Materials

Maleic anhydride (MSA, Merck, Darmstadt, Germany, for synthesis) was sublimed under reduced pressure (50 °C, 2.4 × 10^−2^ bar), benzoyl peroxide (BPO, Merck, for synthesis) was recrystallized from a chloroform:methanol mixture (1:5 vol:vol), 2-butanone (MEK, Merck, p.a.), and other solvents (technical grade) were dried over calcium hydride (Fluka, Seelze, Germany) and distilled before use. 4-methyl-1-pentene (Aldrich, Darmstadt, Germany, for synthesis), methyl iodide (ABCR, Karlsruhe, Germany), benzyl alcohol (Merck, for synthesis), dimethyl sulfoxide (Fluka, p.a.), acetic anhydride (Merck, for synthesis), 3-dimethylaminopropylamine (Aldrich), succinic anhydride (Merck, p.a.), triethylamine (Fluka, p.a.), and anhydrous sodium acetate (Merck, p.a.) were used without additional purification. The inorganic salts sodium chloride, sodium dihydrogen phosphate monohydrate, and disodium hydrogen phosphate dehydrate (all Merck) were used as received.

*Bacteria* have been provided by the German Resource Centre for Biological Material.

(Leibniz-Institut DSMZ-Deutsche Sammlung von Mikroorganismen und Zellkulturen GmbH, Braunschweig, Germany) and (LGC Standards GmbH, Wesel, Germany). Gram-negative bacteria: *Escherichia coli* (DSM 498, ATCC 23716) and *Pseudomonas aeruginosa* (DSM 1117, ATCC 27853).

Gram-positive bacteria: *Staphylococcus aureus* (ATCC 6538) and *Staphylococcus epidermidis* (DSM 1798, ATCC 12228).

Nutrient solutions: (1) NL1*:* 0.5 g of meat extract (Merck) and 0.3 g of peptone obtained from casein (Merck) were dissolved in 99.2 g of sterile water; (2) Mueller-Hinton Broth (Carl Roth GmbH, Karlsruhe, Germany) per L: 2.0 g Beef Extract Powder, 17.5 g Acid Digest of Casein, 1.5 g Starch pH 7.4 ± 0.2.

Phosphate buffered saline (PBS): 100 mL of phosphate buffer and 1.8 g of sodium chloride were placed in a 200-mL measuring flask and filled with distilled water *ad* 200 mL.

### 3.2. Measurements/Apparatus

Size exclusion chromatography was performed using a system consisting of an LC 1120 pump (Polymer Laboratories, Church Stretton, UK), a UV detector ERC-7215, an RI detector ERC-7515A (ERMA CR INC., Kawaguchi, Japan),), a precolumn (50 × 8 mm) of nominal pore size 50 Å, and four columns (300 × 8 mm) filled with MZ-Gel SDplus of nominal pore size 50, 100, 1000, and 10,000 Å (MZ-Analysentechnik, Mainz, Germany). The set was calibrated with PMMA and PS standards from Polymer Laboratories. The sample concentration was 7 mg of polymer in 1 mL of tetrahydrofuran, and the injected volume of the sample was 100 μL. The tetrahydrofuran was stabilized with 2,6-di-tert-butyl-4-methylphenol (250 mg/L).

^1^H-NMR spectra were obtained on a Bruker DPX-300 spectrometer in acetone-d_6_ and D_2_O at 300 MHz. MestRe-C 4.9.0.0 was used as evaluation software. The solvent peak as the reference signal was used.

IR spectra were measured with on an FTIR Thermo Nicolet Nexus spectrometer in KBr-pellets with a resolution of 4 cm^−1^.

Thermogravimetric analysis (TGA) was performed by means of NETZSCH TG 209c thermo balance under nitrogen atmosphere at a nitrogen flow of 15 mL/min. Samples of 9–11 mg were placed in a standard NETZSCH alumina 85-μL crucible. The heating rate was 10 K min^−1^.

Differential scanning calorimetry (DSC) measurements were performed by means of a Netzsch DSC 204 unit. Samples (typical weight: ~9 mg) were enclosed in standard Netzsch 25-μL aluminium crucibles. Indium and palmitic acid were used as calibration standards. Heating and cooling rates were 10 °C min^−1^.

CHN elemental analysis was performed by means of a Carlo Erba MOD-1106 elemental analysis apparatus at the Institute of Organic Chemistry of the RWTH Aachen. Each measurement was performed twice. 

The optical density measurements were performed by means of an Infinite 200 Pro (Tecan, Männedorf, Switzerland) Multiwell plate Reader/Incubator at a wavelength of 612 nm in cycles of 30 min for 20 h.

### 3.3. Syntheses

#### 3.3.1. Poly[(4-methyl-1-pentene)-alt-maleic anhydride] (Copolymer 1, C1)

In a 100-mL round-bottomed flask equipped with a reflux condenser and a valve, maleic anhydride (7 g, 71 mmol, 55 mol %) and 4-methyl-1-pentene (5.04 g 60 mmol) were dissolved in dry MEK (35 mL). To this mixture, BPO (0.3 g, 1.23 mmol, 0.93 mol %) was added and the mixture was degassed three times by freeze–pump–thaw cycles and filled with nitrogen. The reaction mixture was placed in an oil bath at 80 °C for 8 h. After the given time, the reaction mixture was cooled to ambient temperature and the polymer was precipitated in a mixture of diethylether:methanol (4:1 vol:vol), separated by filtration, and dried under vacuum at 40 °C for 8 h. The copolymer (9.3 g, 77% yield) was obtained as a white solid. The molecular weight of the obtained copolymer was determined by gel permeation chromatography in tetrahydrofuran THF-GPC using PMMA standard: M_n_ = 5800, Đ = M_w_/M_n_ = 1.67. The characteristic absorption peaks of IR spectra were: 1852, 1777 (C=O); 927 (C-O-C) cm^−1^. Elemental analysis for C_10_H_14_O_3_; calculated: C 65.9; H 7.7; O 26.4 wt %, found: C 65.54; H 7.85; O 26.61 wt %. ^1^H-NMR (acetone-*d*_6_, δ in ppm): 0.71–4.03; (m, backbone, and side chains)

#### 3.3.2. Determination of the Maleic Anhydride Content of C1 by ^1^H-NMR

C1 (100 mg) was dissolved in 2-butanone (5 mL) and benzyl alcohol (0.5 g) was added. The mixture was heated at 50 °C under reflux for 18 h. The product was precipitated in a diethylether/methanol (1:1) mixture and dried under vacuum at 40 °C for 8 h. ^1^H-NMR (CDCl_3_, δ in ppm): 7.16–7.49 (m, 5H, aromatic); 5.11 (2H, -O-CH_2_-); 0.71–4.03 (m, 14H, backbone, and side chains)

#### 3.3.3. Amidoacidification of C1 to Poly[(4-methyl-1-pentene)-alt-(1-(3-*N*,*N*-dimethylaminopropyl)malemic acid)] (Copolymer 2, C2)

C1 (2.0 g) was dissolved in dry dimethylformamid (DMF) (45 mL) and -(dimethylamino)-1-propylamine (DMAPA) (1.19 g, 11.7 mmol) was added dropwise for 2 h to the stirred solution using a syringe pump at room temperature (RT). During the addition phase, precipitation of the product occurred. The mixture was stirred for an additional 24 h, and the precipitated product, a cream-colored solid, was filtered off and dried at 40 °C under reduced pressure for 8 h. The characteristic absorption peaks of IR spectra were: 1714; 1657; 1567 cm^−1^.

#### 3.3.4. Thermal Imidization of C2 to Poly[(4-methyl-1-pentene)-alt-(1-(3-*N*,*N*-dimethylaminopropyl)maleimide)] (Copolymer 3a, C3a)

Amidoacidified C2 (1.0 g) was dissolved in DMF (20 mL) and heated under reflux at 120 °C for 24 h. The product was precipitated from diethylether and dried under vacuum at 40 °C for 5 h. The molecular weight of the obtained copolymer was determined by THF-GPC using PMMA standard: M_n_ = 8600, Đ = 1.46. The characteristic absorption peaks of IR spectra were: 1772; 1697 cm^−1^. Elemental analysis for C_15_H_26_O_2_N_2_; calculated: C 67.7; H 9.8; N 10.5; O 12.0 wt %; found: C 67.09; H 9.70; N 10.11; O 11.10 wt %.

#### 3.3.5. Chemical Imidization of Amidoacidified C2 (Copolymer 3b, C3b)

The amidoacidified C2 (0.5 g) was dissolved in a mixture of DMF, acetic anhydride, 2-butanone triethylamine, or sodium acetate (see Table 6 for detailed composition). The reaction mixture was either heated under reflux or stirred at RT under a protective atmosphere of nitrogen for 12–18 h. The product was precipitated in diethylether as a dark-brown solid and dried under vacuum at 40° for 24 h. The repeated precipitation had no influence on the appearance. The characteristic absorption peaks of IR spectra were: 1770; 1697 cm^−1^.

#### 3.3.6. Preparation of Imide C3 in a One-Pot Reaction (Copolymer 3c, C3c)

C1 (3.5 g) was dissolved in dry DMF (20 mL) and placed in a 250-mL round-bottomed flask. Within 2 h, DMAPA (2.5 g) dissolved in dry DMF (40 mL) was added by means of a syringe pump at room temperature. The dispersion of the precipitated product was stirred additionally for 12 h and the mixture was placed in an oil bath heated to 120 °C for another 24 h. The product was purified by precipitation in 400 mL of diethylether. After drying the precipitate under vacuum at 40 °C for 8 h, a cream-colored powder was obtained (3.2 g, 97.5%). The molecular weight of the obtained copolymer was determined by THF-GPC using PMMA standard: M_n_ = 9350, Đ = 1.40. The characteristic absorption peaks of IR spectra were: 1772; 1697 cm^−1^. Elemental analysis for C_15_H_26_O_2_N_2_; calculated: C 67.7; H 9.8; N 10.5; O 12.0 wt %; found: C 67.16; H 9.76; N 10.21; O 12.87 wt %.

#### 3.3.7. Synthesis of Poly[(4-methyl-1-pentene)-alt-(1-(3-*N*,*N*,*N*-trimethylammonium-propyl)-maleimidoiodide)] by Quaternization of Imidized C3 with Methyl Iodide (Copolymer 4, C4)

Thermally imidized C3a (0.1 g) was dissolved in dimethyl sulfoxide DMSO (4 mL) and placed in a 25-mL round-bottomed flask. Methyl iodide (0.1 mL) was added and the mixture was stirred at room temperature for 18 h. The product was precipitated in THF and dried under vacuum at 40 °C for 12 h. The product (0.147 g, 96%) was obtained as a lemon-yellowish powder. Elemental analysis for C_16_H_29_O_2_N_2_I; calculated: C 47.1; H 7.2; N 6.9; O 7.6; I 31.2 wt %; found: C 46.63; H 7.0; N 6.97; O 7.87 wt %.

#### 3.3.8. Sequential Quaternization of Imidized C3 with Methyl and Dodecyl Iodide (Copolymer 5, C5)

Thermally imidized C3a (0.3 g) was placed in a 25-mL round-bottomed flask and dissolved in a mixture of acetone (10 mL) and DMSO (5 mL). Dodecyl iodide (0.164 g, 0.564 mmol, 50% with respect to the *N*,*N*-dimethylammonium groups) of) was added and the mixture was stirred for 48 h. In a subsequent step, methyl iodide (0.2 g, 1.4 mmol) dissolved in DMSO (5 mL) was added and the reaction mixture was stirred for another 24 h. The product was precipitated in a THF/hexane (4:1) mixture, separated, and dried under vacuum at 40 °C for 8 h. Elemental analysis for C_43_H_80_O_4_N_4_I_2_; calculated: C 53.2; H8.2; N5.8; O 6.6; I 26.2 wt %; found: C 53.0; H 8.17; N 5.84; O 6.9 wt %.

#### 3.3.9. Synthesis of Poly[(4-methyl-1-pentene)-alt-(1-(3-*N*,*N*-dimethyl-*N*-dodecylammoniumpropyl)-maleimidoiodide)] (Copolymer 6, C6)

Thermally imidized C3a (0.1g) was dissolved in acetone (4 mL) and placed in a 25-mL round-bottomed flask and dodecyl iodide (0.1 mL) was added. The flask was closed with a glass stopper and the mixture was stirred for 48 h. The product was precipitated in hexane, filtered, and dried under vacuum at 40 °C for 8 h. Elemental analysis for C_27_H_51_O_2_N_2_I; calculated: C 57.6; H 9.0; N 5.0; O 5.7; I 22.7 wt %; found: C 57.56; H 9.06; N 5.01; O 5.88 wt %.

#### 3.3.10. Synthesis of 3-(*N*,*N*-dimethylamino)propyl Maleic Acid Amide (M1)

Maleic anhydride (7.0 g, 71 mmol) was dissolved in dry chloroform (100 mL) and placed in a 250-mL flask. A solution of DMAPA (6.8 g, 67 mmol) in chloroform (20 mL) was added dropwise and stirred for 1 h at 60 °C. After the addition period, the reaction mixture was heated at 60 °C for 1 h. The product was precipitated in a fivefold excess of acetone (with respect to the volume of the reaction mixture), separated, and dried under vacuum at 40 °C for 8 h. The product (12.2 g, 88%) was obtained as a white powder. The characteristic absorption peaks of IR spectra were: 1710, 1650, 1588 cm^−1^. ^1^H-NMR (D_2_O, δ in ppm): 6.33 (dd, 1H, C = CH-CON-, ^3^J = 12 Hz); 5.96 (dd, 1H, C = CH-COOH, ^3^J = 12 Hz); 3.33 (t, 2H, -NH-CH_2_-CH_2_-CH_2_-N(CH_3_)_2_, ^3^J = 6 Hz); 3,17 (t, 2H, -NH-CH_2_-CH_2_-CH_2_-N(CH_3_)_2_, ^3^J = 9 Hz); 2,88 (s, 6H, -NH-CH_2_-CH_2_-CH_2_N-(CH_3_)_2_); 1,95 ( q, 2H, -NH-CH_2_-CH_2_-CH_2_N-(CH_3_)_2_) ^3^J = 6 Hz).

#### 3.3.11. Chemical Imidization (Z)-4-(*N*,*N*-dimethylamino)propylamino-4-oxobut-2-enoic Acid (M2)

with a reflux condenser for 4 h at 90 °C. After cooling to ambient temperature, the reaction mixture Maleamic acid (1.0 g, 5 mmol) and sodium acetate (0.2 g, 2 mmol) were dissolved in acetic anhydride (30 mL) and heated in a 50-mL round-bottomed flask equipped was filtered to remove sodium acetate, and acetic anhydride was distilled off under reduced pressure. A brown oily product (0.8 g, 72%) was obtained. The characteristic absorption peaks of the IR spectra were: 1773; 1697 cm^−1^. ^1^H-NMR (D_2_O, δ in ppm): 6.86 (s, 2H, -OC-CH = CH-CO-); 3.62 (t, 2H, -NH-CH_2_-CH_2_-CH_2_-N(CH_3_)_2_, ^3^J = 6 Hz); 3.14 (t, 2H, -NH-CH_2_-CH_2_-CH_2_-N(CH_3_)_2_, ^3^J = 9 Hz); 2.86 (s, 6H, -NH-CH_2_-CH_2_-CH_2_N-(CH_3_)_2_); 1.98 (q, 2H, -NH-CH_2_-CH_2_-CH_2_N-(CH_3_)_2_) ^3^J = 6 Hz).

#### 3.3.12. Synthesis of 3-(*N*,*N*-dimethylamino)propyl Succinamic Acid (M3)

DMAPA (3.6 g, 35 mmol) was added slowly to a solution of succinic anhydride 3.5 g (35 mmol) in dry acetone (20 mL). The mixture was stirred for 2 h at room temperature. The precipitated product was filtered and immediately used for the thermal imidization reaction without further characterization.

#### 3.3.13. Thermal Imidization of (*Z*)-4-(*N*,*N*-dimethylamino)propylamino-4-oxobutanoic Acid (M4)

3-(*N*,*N*-dimethylamino)propyl succinamic acid obtained from the reaction of succinic anhydride and DMAPA was heated under flowing nitrogen at 170 °C for 2 h. A brownish oily liquid (6.3 g, 98%) was obtained. The characteristic absorption peaks of IR spectra were: 1770; 1697 cm^−1^. ^1^H-NMR (D_2_O, δ in ppm): 3.23 (t, 2H, -NH-CH_2_-CH_2_-CH_2_-N(CH_3_)_2_, ^3^J = 6 Hz); 2.97 (t, 2H, -NH-CH_2_-CH_2_-CH_2_-N(CH_3_)_2_, ^3^J = 6 Hz); 2.73 (s, 6H, -NH-CH_2_-CH_2_-CH_2_N-(CH_3_)_2_); 2.41 (s, 4H, -OC-CH_2_-CH_2_-CO-); 1.85 (q, 2H, -NH-CH_2_-CH_2_-CH_2_N-(CH_3_)_2_.

### 3.4. Antimicrobial Tests

The antibacterial activity of the amphiphilic polymers in solution was determined by measuring the minimum inhibitory concentration (MIC) using the test bacteria mentioned above. Suspensions of strains with known colony forming units (CFU; 2 × 10^6^ CFU/mL) were incubated at 37 °C in nutrient solution (Mueller-Hinton Broth, MHB) with different concentrations of the polymer samples. The polymer samples were solubilized in bidistilled water and added to the nutrient solution at a constant ratio of 1:10. The growth of the bacteria was followed during the incubation over 20 h by measuring the optical density at 612 nm every 30 min (with 1000 s of shaking at 100 rpm per 30 min cycle by using a microwell plate reader/incubator (TECAN Infinite 200 Pro, Tecan Trading AG, Männedorf, Switzerland). The testing was performed with defined concentrations specifically for each polymer until, within the monitoring time of 20 h, no bacterial growth curve was recorded. All experiments were performed in triplicate duplicates and MIC determination was repeated on three different days. The polymers were not sterilized. Sterile controls (defined polymer concentrations in nutrient solution without bacteria) were assessed in every growth curve monitoring testing series. MICs were determined according to broth microdilution in 96-well microtitre plates [76]. The MIC corresponds to the concentration of the test substance at which a complete inhibition of the growth of the inoculated bacteria was observed by comparison with control samples without test substance.

### 3.5. Hemolytic Activity

Hemolytic activity was assessed according to the literature [77]. Human erythrocytes (from healthy donors, red blood cells (RBC), 0, Rh positive, citrate-phosphate-dextrose-adenine-stabilized; CPDA1 Sarstedt Germany) were obtained by centrifugation (3500 rpm, 12 min) to remove plasma, washed three times in PBS (0.01 M phosphate buffered saline, Sigma Aldrich Chemie GmbH, Steinheim, Germany), and diluted in PBS to obtain a stock solution of 2.5 × 10^8^–3.0 × 10^8^/mL RBC. Solutions of defined polymer concentration (250 µL) were pipetted into 250 μL of the stock solution, the final amount of RBC being 1.2 × 10^8^–1.5 × 10^8^ RBC/mL. The RBC were exposed for 60 min at 37 °C under 3D-shaking, centrifuged thereafter (4000 rpm, 12 min), and the absorption of the supernatant (diluted 10-fold in PBS) was determined at 414 nm in a microplate reader. As reference solutions, (i) PBS for determining spontaneous hemolysis and (ii) 1% Triton X-100 for 100% hemolysis (positive control) were used. Hemolysis was plotted as a function of polymer concentration and the hemolytic activity was defined as the polymer concentration that causes 50% hemolysis of human RBC relative to the positive control (HC_50_).

## 4. Conclusions

Maleic anhydride copolymers are versatile, easy-for-modification materials which can be used as a base for a wide range of antimicrobial copolymers.

The modification of P[MP-alt-MSA] copolymer with diamine to poly[(4-methyl-1-pentene)-alt-(1-(3-*N*,*N*-dimethylaminopropyl)maleimide)] can be performed as a one-pot synthesis without using any additives in relatively mild conditions. Poly[(4-methyl-1-pentene)-alt-(1-(3-*N*,*N*-dimethylaminopropyl)maleimide)] can be easily converted into a polycationic material by means of any alkyl iodide. Sequential quaternization with methyl iodide and dodecyl iodide—which introduce a hydrophobic long-alkyl-chain moiety, but thanks to methyl iodide, ensure solubility in polar solvents—shows the best properties in the sense of antimicrobial activity.

The antimicrobial properties of poly[(4-methyl-1-pentene)-alt-(1-(3-*N*,*N*,*N*-trimethylammoniumpropyl)-maleimidoiodide)] are even lower than the properties of the nonquaternized copolymer.

The polymers are more effective by a factor of 5–10 against the Gram-negative *E. coli* than against the Gram-positive *S. aureus*. Compared to poly[(4-methyl-1-pentene)-alt-(1-(3-*N*,*N*,*N*-trimethylammoniumpropyl)-maleimidoiodide)], polymers poly[(4-methyl-1-pentene)-alt-(1-(3-*N*,*N*-dimethylaminopropyl)maleimide)] and poly[(1-(3-*N*,*N*,*N*-trimethylammonium-propyl)-maleimidoiodide)-co-(1-(3-*N*,*N*-dimethyl-*N*-dodecylammoniumpropyl) maleimidoiodide)-alt-(4-methyl-1-penten)] are more active against the Gram-negative bacteria *E. coli* and *P. aeruginosa*, whereas, in the case of *S. aureus*, only poly[(1-(3-*N*,*N*,*N*-trimethylammonium-propyl)-maleimidoiodide)-co-(1-(3-*N*,*N*-dimethyl-*N*-dodecylammoniumpropyl) maleimidoiodide)-alt-(4-methyl-1-penten)] was the most active polymer compared to the nonquaternized polymer and the polymer quaternized with methyl iodide. The nonquaternized copolymer was the least active in the latter case.

The MIC of the synthetic copolymers is higher than of the natural peptide Nisin. However, the freedom in designing the basic polymer, molecular weight, as well as the way of modification and, in particular, the much lower price of the synthetic compounds show the potential of the presented strategy to develop new “surface protection”.

## Figures and Tables

**Table 1:** Comparison of the experimental and calculated elemental composition of an alternating copolymer poly[(4-methyl-1-pentene)-alt-maleic anhydride] (C_10_H_14_O_3_).

Composition	Carbon	Hydrogen	Oxygen
Calculated for C,H,O	65.90	7.70	26.40
Found	65.54	7.85	26.61

**Table 2:** Comparison of the CHN elemental analysis results of the amidoacidified copolymer with calculated values.

#	Composition	Carbon	Hydrogen	Nitrogen
#1	Calculated for C_15_H_28_N_2_O_3_	64.30	9.80	9.80
#2	Calculated for C_15_H_28_N_2_O_3_·2H_2_O	56.23	10.07	8.74
#3	Found	56.18	9.73	9.78

**Table 3:** Solubility of imidized C3 and quaternized C4, C5, and C6 in chosen solvents (10 mg of copolymer/mL). (+) well soluble (>10 wt %); (−) nonsoluble (<1 wt %).

Solvent	C3	C4	C5	C6
Water	+	+	+	−
Methanol	+	−	+	−
Ethanol	+	−	+	−
Acetone	+	−	+	+
2-Butanone	+	−	+	+
Ethyl acetate	+	−	−	−
THF	+	−	+	+
DMSO	+	+	+	−
DMF	+	+	+	−
Chloroform	+	−	−	+

THF = tetrahydro furane, DMSO = dimethyl sulfoxide, DMF = dimethylformamide.

**Table 4:** Transition temperatures for nonquaternized C3 and its cationic modifications.

#	Transition 1 (°C)	Transition 2 (°C)	Functional Group
C3	18	88	-N(CH_3_)_2_
C4	17	85	-N(CH_3_)_3_^+^
C5	17	104	-N(CH_3_)_3_^+^ + -N(CH_3_)_2_ C_12_H_25_^+^
C6	18	101	-N(CH_3_)_2_ C_12_H_25_^+^

**Table 5:** Minimum inhibitory concentration (MIC) of the polymeric materials in bidistilled water and Mueller-Hinton broth (MHB) against bacteria (1–2 × 10^6^ cfu/mL) (MIC_100_: complete growth inhibition during the monitoring time of 20 h) compared to reference Nisin, and hemolytic effect (HC_50_).

Polymer	MIC_100_ in µg/mL				HC_50_ in µg/mL
	*E. coli ATCC 23716*	*P. aeruginosa ATCC27853*	*S. aureus ATCC 6538*	*S. epidermidis ATCC 12228*	
C3	20	100	>1000	20	100 *
C4	200	>>1000	1000	20	>>1000 **
C5	10	200	100	20	60 ^#^
NISIN	100	>200	3	3	>>340 ^+^

* Agglutination of red blood cells (RBC) at all concentrations tested 10–1000 µg/mL; ** agglutination of RBC at 20–1000 µg/mL; # no agglutination of RBC, + at 340 µg/mL = 100 mM Nisin only 10.8% of the RBC are lysed. Nisin (Handary 97.9%).

**Table 6:** Composition of the reaction mixtures, reaction conditions for the chemical imidization of C2, yield, and appearance of the product.

#	DMF (g)	Ac_2_O (g)	MEK (g)	Et_3_N (g)	CH_3_COONa (g)	T (°C)	t (h)	Yield %	Appearance
1	15	15	-	0.1	-	RT	12	88	brown powder
2	15	15	-	0.1	-	80	18	70	brown powder
3	20	1.07	-	1.41	-	80	24	62	brown, tarry
4	-	1.0	15	0.8	-	90	18	87	grey powder
5	-	0.55	15	0.73	-	90	12	90	grey powder
6	-	7	-	1	-	80	20	78	grey powder
7	-	10	-	-	0.1	80	18	69	brown powder

DMF = dimethylformamid, Ac_2_O = acetic anhydride, MEK = 2-butanone, Et_3_N = triethylamine, CH_3_COONa = sodium acetate, RT = room temperature.
